# Does Vitamin D Sufficiency Equate to a Single Serum 25-Hydroxyvitamin D Level or Are Different Levels Required for Non-Skeletal Diseases?

**DOI:** 10.3390/nu5125127

**Published:** 2013-12-16

**Authors:** Simon Spedding, Simon Vanlint, Howard Morris, Robert Scragg

**Affiliations:** 1 Division of Health Sciences, University of South Australia, Adelaide, SA 5000, Australia; E-Mails: spedding@adam.com.au (S.S.); howard.morris@health.sa.gov.au (H.M.); 2 Discipline of General Practice, School of Population Health, University of Adelaide, Adelaide, SA 5005, Australia; E-Mail: simon.vanlint@adelaide.edu.au; 3 SA Pathology, PO Box 14, Rundle Mall, Adelaide, SA 5000, Australia; 4 School of Population Health, Faculty of Medical and Health Sciences, The University of Auckland, Private Bag 92019, Auckland 1142, New Zealand; E-Mail: r.scragg@auckland.ac.nz

**Keywords:** vitamin D, dose response, thresholds, prevention, chronic disease

## Abstract

Objective: Clarify the concept of vitamin D sufficiency, the relationship between efficacy and vitamin D status and the role of Vitamin D supplementation in the management of non-skeletal diseases. We outline reasons for anticipating different serum vitamin D levels are required for different diseases. Method: Review the literature for evidence of efficacy of supplementation and minimum effective 25-hydroxyvitamin D (25-OHD) levels in non-skeletal disease. Results: Evidence of efficacy of vitamin supplementation is graded according to levels of evidence. Minimum effective serum 25-OHD levels are lower for skeletal disease, e.g., rickets (25 nmol/L), osteoporosis and fractures (50 nmol/L), than for premature mortality (75 nmol/L) or non-skeletal diseases, e.g., depression (75 nmol/L), diabetes and cardiovascular disease (80 nmol/L), falls and respiratory infections (95 nmol/L) and cancer (100 nmol/L). Conclusions: Evidence for the efficacy of vitamin D supplementation at serum 25-OHD levels ranging from 25 to 100 nmol/L has been obtained from trials with vitamin D interventions that change vitamin D status by increasing serum 25-OHD to a level consistent with sufficiency for that disease. This evidence supports the hypothesis that just as vitamin D metabolism is tissue dependent, so the serum levels of 25-OHD signifying deficiency or sufficiency are disease dependent.

## 1. Introduction

Vitamin D supplementation is established in both the prevention and treatment of bone disease [1] but its role in other clinical situations remains contentious. This article examines the role of vitamin D in the management of non-skeletal disease.

The term “vitamin D” is a misnomer. It is a secosteroid pro-hormone synthesized in response to sunshine exposure and has autocrine, paracrine and endocrine modes of action [2]. Vitamin D receptors (VDRs) occupy 2776 genomic positions and modulate 229 genes [3]. The wide distribution of VDRs suggests a role for vitamin D in most physiological systems of the body, implying that deficiency may lead to physiological changes predisposing to chronic diseases. Vitamin D deficiency is a public health concern as it is pandemic [4], affecting a third of the population, even in tropical and subtropical regions [5,6,7,8,9,10]. There is considerable debate about terminology and serum 25-hydroxyvitamin D (25-OHD) levels [11]. A 25-OHD concentration for vitamin D deficiency is defined as <50 nmol/L and sufficiency as >75 nmol/L in this paper, although the concept of a single level for sufficiency is not universal, one example being acetylsalicylic acid that requires different serum levels for analgesia and anticoagulation.

There is substantial disagreement about what constitutes vitamin D sufficiency and the role of vitamin D in the management of non-skeletal disease, despite an exponential increase in vitamin D research and myriad of consensus statements and guidelines [12,13]. Thus clinicians still lack clear information about efficacy of vitamin D and the relationship between supplement dose or vitamin D status and clinical response in non-skeletal disease.

Whilst the influential Institute of Medicine (IOM) report on calcium and vitamin D asserted that the role for vitamin D was exclusive to bone health, based on null studies relating to non-skeletal disease [1], more than 12,000 vitamin D articles have been published since the IOM report [12]. This suggests it is timely to review the role of vitamin D in non-skeletal disease. Furthermore the view is gaining traction that these null studies relating to non-skeletal disease were due to methodological limitations of inadequate doses of vitamin D and inappropriate baseline levels of 25-OHD that led to inept conclusions about vitamin D efficacy [13,14]. Studies and reviews with these limitations were identified using the approach advocated by Lappe and Heaney [15]. These include studies that did not measure baseline 25OHD levels [16] or used doses of vitamin D that were unlikely to achieve sufficiency [16,17] or whose participants who were already vitamin D replete [18,19].

## 2. Tissue Dependence of Vitamin D

These design limitations have a profound effect on the results due to the pharmacokinetics of vitamin D. Unlike most vitamins and many nutrients, vitamin D activity is tissue-dependent. Different levels of 25-OHD have different therapeutic effects which vary according to the target tissue [2]. 25-OHD is not the active form of vitamin D and requires hydroxylation to the active form 1,25-dihydroxyvitamin D (1,25OH_2_D). This occurs either in the kidney, which accounts for circulating concentrations of 1,25OH_2_D, and locally in specific tissues through the action of the enzyme 25-hydroxyvitaminD_3_-1α-hydroxylase. However the levels of this enzyme vary between tissues, therefore the serum 25-OHD concentration required for the adequate synthesis of 1,25OH_2_D also varies between tissues [20].

It follows that each physiological or therapeutic effect may require a specific minimum serum 25-OHD concentration. Good evidence already exists for differential levels in bone disease, with most evidence suggesting that serum 25-OHD concentrations of at least 20–25 nmol/L are required to prevent rickets, but that higher concentrations of 50–60 nmol/L are required to prevent osteoporosis and fractures [21].

We explore the evidence for therapeutic levels in non-skeletal disease, and hypothesise that tissue dependence may be responsible for the limitations of some studies included in previous reviews.

## 3. Aims of This Paper

1. To examine trials and reviews of vitamin D supplementation in non-skeletal diseases.
2. To examine the minimum effective levels of 25-OHD that are associated with non-skeletal diseases.
3. To assess the level of evidence for the efficacy of vitamin D supplementation in non-skeletal disease.

## 4. Methods

This review focuses on studies with a primary endpoint of a non-skeletal disease using therapeutic doses that were shown to improve participants’ vitamin D status. Studies were collected through Medline searches and by examining the publications of two US groups: the IOM [1] and the Vitamin D Council [12].

The authors reviewed evidence for vitamin D in the management of non-skeletal diseases. Studies and reviews with significant limitations due to design flaws were identified, using the approach advocated by Lappe and Heaney [15], which emphasises the value of RCTs, where baseline vitamin D status was known and where the supplemental dose of vitamin D administered could therefore be shown to have improved vitamin D status. However, excluding studies which did not satisfy all of these criteria was not always possible due to limitations of the current evidence base.

Consensus statements will be presented for:

1. The highest level of evidence for each non-skeletal disease, evaluated according to the Australian National Health and Medical Research Council levels of evidence (Table 1) [22];
2. The minimum effective serum level of 25-OHD in non-skeletal disease.

**Table 1:** Australian National Health and Medical Research Council (Australia) Evidence Hierarchy: designations of “levels of evidence” [22].

Level	Intervention
**I**	Systematic review of Level II studies
**II**	Randomised controlled trial
**III-1**	Pseudo-randomised controlled trial
**III-2**	Comparative study with concurrent controls: non-randomised, experimental trial, cohort study, case-control study, or interrupted time series with a control group
**III-3**	A comparative study without concurrent controls: historical control study, or
	two or more single arm study, Interrupted time series without a parallel control group
**IV**	Case series with either post-test or pre-test/post-test outcomes

## 5. Findings

**Premature mortality**—There is Level I evidence that vitamin D supplementation reduces premature mortality. A Cochrane review of randomized controlled trials (RCTs) showed a 6% reduction in all-cause mortality [23], with observational data from the NHANES and ESTHER studies suggesting a minimum 25-OHD concentration of 75 nmol/L for preventing premature mortality [24,25].

**Cancer**—There is Level II evidence from one RCT that vitamin D supplementation leads to a substantial reduction in overall risk of internal cancers [26]. The reported minimum 25-OHD concentration for cancer prevention is 100 nmol/L [27] whilst a review shows an inverse, non-linear relationship between 25-OHD levels and cancer risk [28]. 

**Muscle function and falls**—There is Level I evidence from a meta-analysis that demonstrated a 26% reduction in falls with vitamin D supplementation [29]. Other meta-analyses [30,31,32] that included RCTs with design limitations reached a null conclusion [33]. The optimal 25-OHD concentration for lowest risk of falls is 95 nmol/L [34].

There is Level II evidence for the efficacy of vitamin D supplementation in vitamin D deficiency for lower limb muscle strength [35].

**Cardiovascular disease**—There is Level III-2 evidence that vitamin D protects against cardiovascular disease from three meta-analyses of cohort studies [36,37]. One study of 300,000 person years demonstrated a 39% reduction in IHD in people with high 25-OHD concentrations in comparison with those with severe deficiency and a similar effect on incidence of ischaemic stroke [38]. In another study, Vitamin D supplementation conferred substantial survival benefit (OR for death 0.39 *p* < 0.0001) in cardiac patients [39]. These estimates are very similar to those proposed in an earlier systematic review and meta-analysis by Parker *et al*. [40]. The reported minimum 25-OHD concentration associated with lowest CVD risk is 80 nmol/L [41].

**Respiratory infections**—There is Level II evidence that vitamin D supplementation reduces rates of respiratory infections including pneumonia [42], influenza [43] and upper respiratory infections [44]. This is supported by epidemiological studies for tuberculosis [45,46] and other respiratory infections [47,48,49]. The reported minimum 25OH-D concentration associated with a reduced incidence of viral respiratory infections is 95 nmol/L [50].

**Diabetes**—There is Level II evidence from two randomized controlled trials that vitamin D supplementation improves insulin sensitivity and decreases insulin resistance [51,52]. This is supported by three meta-analysis of cohort studies, which found low 25-OHD concentrations predicted increased risk of type 2 diabetes [53,54,55]. The reported minimum vitamin D concentration for reducing insulin resistance is 80 nmol/L [56].

**Depression**—There is Level II evidence that vitamin D supplementation is effective in treating depression from four RCTs in which supplementation significantly reduced depression in vitamin D deficient depressed patients [56,57,58,59]. The reported minimum 25OH-D concentration associated with reduced depression is 95 nmol/L [58].

**Dental disease**—There is Level II evidence from a RCT for dental caries [60]. There is also evidence from the NHANES database showing that attachment loss (AL) is significantly and inversely correlated with serum 25-OHD concentrations in those aged over 50 years AL was lowest in individuals whose 25-OHD levels exceeded 84 nmol/L [61].

**Pain**—There is Level II evidence for the efficacy of vitamin D supplementation in chronic musculoskeletal pain patients who are also vitamin D deficient [62]. An RCT in vitamin D insufficient non-Western immigrants in the Netherlands showed a clinically meaningful positive effect on pain 6 weeks after high-dose vitamin D supplementation [63]. No threshold level has been proposed.

**Health service utilisation and costs**—There is Level III-2 evidence that vitamin D deficiency increases health care utilisation and cost. A large study of health care costs in US veterans found that vitamin D deficiency predicted increased health care costs at all six participating sites [64]. Another study estimated that health care utilisation was 39% higher in vitamin D deficient patients [65]. Vitamin D supplementation may be more cost effective than currently funded prevention programs; single studies show that supplementation appears to be more cost effective than screening programs for bowel cancer [66] and more cost effective than multifactorial interventions for preventing falls in the elderly [67]. Considering the economic burden of disease due to vitamin D deficiency, the estimated benefit of increasing vitamin D status across Western Europe was said to be €187b per year [68], calculated on a population basis the equivalent figure for Australia was $14b per year.

## 6. Summary of the Evidence

There is evidence for the efficacy of vitamin D (Table 2):

Level I for prevention of falls and premature mortality;
Level II for prevention of diabetes, respiratory infections, cancer and for managing musculoskeletal pain and depression;
Level III for health service utilisation, prevention of cardiovascular disease, musculoskeletal strength and dental disease.

Evidence supports the contention that specific 25-OHD levels may be required to prevent disease, with higher concentrations required for non-skeletal disease. Table 2 shows the levels associated with lowest risk of the following diseases:

20–25 nmol/L for osteomalacia, and rickets,
50–60 nmol/L for osteoporosis, and fractures,
75–85 nmol/L for premature mortality cardiovascular disease, diabetes, and for treatment of depression and dental disease,
95–100 nmol/L for falls, cancer, and respiratory infections.

**Table 2:** Proposed vitamin D concentrations in the management of non-skeletal diseases.

	*Level of evidence NHMRC* [24]	*Minimum effective serum 25-OHD concentration in nmol/L* [24]
Premature mortality	Level l	75
Falls prevention	Level l	95
Cancer prevention	Level ll	100
Respiratory infection prevention	Level ll	95
Diabetes prevention	Level ll	80
Depression treatment	Level ll	75
Musculoskeletal pain management	Level ll	
Dental disease	Level lll-2	>84
Musculoskeletal strength	Level lll-1	
Cardiovascular disease	Level lll-2	80
Health service utilisation	Level lll-2	

## 7. Discussion

The evidence supports the following conclusions:

- The use of vitamin D supplements in the management of non-skeletal diseases is supported by; High level of evidence for reducing premature mortality, falls (Level I), Moderate evidence for reducing diabetes, respiratory infections, depression and some cancers (Level II), and a Lower level of evidence for preventing cardiovascular disease, and treating musculoskeletal pain and dental disease(Level III).
- The minimum level of 25-OHD required for preventing non-skeletal disease is higher than for skeletal disease.
- Economic evaluations indicate vitamin D supplementation reduces health service costs.

However there is still skepticism about the role of vitamin D in non-skeletal disease based on the IOM report [1]. Since this report there have been 12,000 vitamin D publications, including reviews showing increasing consistency in the evidence about efficacy and the therapeutic levels in non-skeletal disease [69,70,71,72]. This evidence comes from research in different disciplines, study designs, and countries. Beyond this evidence, there are publications showing how the Bradford Hill criteria are satisfied in non-skeletal diseases [73], and genetic evidence from the first randomized trial on gene expression demonstrating a “molecular fingerprint that help explain the non-skeletal health benefits of vitamin D” [74].

There is currently considerable concern regarding the accuracy of serum 25-OHD measurements. While international efforts towards a standardisation program for this assay are making considerable progress, we have worked with the best available evidence that is currently available. We recognize that these ranges of 25–50 nmol/L for skeletal disease and 75–100 nmol/L for non-skeletal disease may change slightly when a standardisation program is fully implemented. However it is likely these diverse ranges for skeletal and non-skeletal disease will remain quite separate even after standardisation. This would be consistent with the hypothesis that the different ranges reflect different mechanisms or tissue dependence, as seen with other medicines, such as acetylsalicylic acid.

It is likely that an improved community vitamin D status would reduce health care utilisation and costs, and thus the economic burden of a wide range of chronic diseases. Adequately-powered, purpose-designed, randomised studies may provide greater certainty if baseline 25OHD is measured and adequate doses of vitamin D are used. Currently clinicians may accept the information provided pathology companies as to what constitutes a “target” 25-OHD level. Pathology companies generally suggest a wide target range, for example 60–160 nmol/L rather than specifically designate levels for deficiency and sufficiency, whilst the Royal College of Pathologists of Australasia is less specific, suggesting a level greater than 50 nmol/L for “optimal health”.

There is concern about morbidity at high levels of 25-OHD raised by epidemiological studies, a retrospective cohort in general practice (CopD) [75] and a prospective community cohort study (NHANES III) [76]. They support the hypothesis of a reverse J curve indicating morbidity increases at higher levels of 25-OHD (140 nmol/L). The authors grant that these studies “did not allow inference of causality”. Whereas meta-analysis of prospective cohort studies do provide Level I evidence of causality according to the NHMRC [22]. Three meta-analyses did not demonstrate increased mortality at higher levels of 25OHD [77,78,79]. A 28% reduction in mortality in the highest category was demonstrated in the largest study and largest meta-analysis performed to date [78]. These studies indicate there is conflicting evidence for an upper inflection point in a J shaped curve at “high” 25-OHD levels.

We hypothesise that just as vitamin D metabolism is tissue dependent, so the levels of 25-OHD that signify sufficiency are disease dependent. Thus the response to vitamin D supplementation is dependent on the baseline 25-OHD level, the dose of vitamin D and dose-response characteristics.

In the field of research, trials can be designed to minimise limitations of study design that lead to null outcomes knowing the range of serum 25-OHD in which changes in serum levels will lead to responses in specific body systems. This will also provide more clarity when assessing the effectiveness of vitamin D in different diseases, greater uniformity in the results of meta-analyses and less confusing guidelines.

In the field of health care, knowledge of the minimum therapeutic level for vitamin D will ensure clinicians can provide the correct dose and monitor it appropriately for treatment of specific diseases, and that policy makers have the opportunity to make evidence-based decisions about nutritional advice, supplementation, and fortification of food with vitamin D.

Further research to clarify the specific therapeutic levels for vitamin D sufficiency in different diseases and the dose response characteristics of vitamin D will ensure progress in research, clinical medicine, and public health.

## References

[1] Ross A., Taylor C., Yaktine A., del Valle H. (2010). Dietary reference intakes for calcium and vitamin D.

[2] Morris H., Anderson P. (2010). Autocrine and paracrine actions of vitamin D. Clin. Biochem. Rev..

[3] Ramagopalan S.V., Heger A., Berlanga A.J., Maugeri N.J., Lincoln M.R., Burrell A., Handunnetthi L., Handel A.E., Disanto G., Orton S.-M. (2010). A ChIP-seq defined genome-wide map of vitamin D receptor binding: Associations with disease and evolution. Genome Res..

[4] Holick M. (2010). The vitamin D deficiency pandemic: A forgotten hormone important for health. Pubic Health Rev..

[5] Daly R.M., Gagnon C., Lu Z.X., Magliano D.J., Dunstan D.W., Sikaris K.A., Zimmet P.Z., Ebeling P.R., Shaw J.E. (2012). Prevalence of vitamin D deficiency and its determinants in Australian adults aged 25 years and older: A national, population-based study. Clin. Endocrinol..

[6] Van der Mei I., Ponsonby A., Engelsen O., Pasco J., McGrath J., Eyles D., Blizzard L., Dwyer T., Lucas R., Jones G. (2007). The high prevalence of vitamin D insufficiency across Australian populations is only partly explained by season and latitude. Environ. Health Perspect..

[7] Sud S.R., Montenegro-Bethancourt G., Bermúdez O.I., Heaney R.P., Armas L., Solomons N.W. (2010). Older Mayan residents of the western highlands of Guatemala lack sufficient levels of vitamin D. Nutr. Res..

[8] Sheikh A., Saeed Z., Jafri S.A.D., Yazdani I., Hussain S.A. (2012). Vitamin D levels in asymptomatic adults—A population survey in Karachi, Pakistan. PLoS One.

[9] Arabi A., El Rassi R., El-Hajj Fuleihan G. (2010). Hypovitaminosis D in developing countries—Prevalence, risk factors and outcomes. Nat. Rev. Endocrinol..

[10] Lips P. (2010). Worldwide status of vitamin D nutrition. J. Steroid Biochem. Mol. Biol..

[11] Scragg R. (2011). Vitamin D and public health: An overview of recent research on common diseases and mortality in adulthood. Pubic Health Nutr..

[12] Exponential Growth in Vitamin D Publications. http://www.vitamindwiki.com/tiki-index.php?page=Expodential+growth+in+vitamin+D+publications.

[13] Heaney R. (2012). Vitamin D—Baseline status and effective dose. N. Engl. J. Med..

[14] Heaney R. (2008). Nutrients, endpoints, and the problem of proof. J. Nutr..

[15] Lappe J., Heaney R. (2012). Why randomized controlled trials of calcium and vitamin D sometimes fail. Dermato-Endocrinology.

[16] Jackson R.D., LaCroix A.Z., Gass M., Wallace R.B., Robbins J., Lewis C.E., Bassford T., Beresford S.A.A., Black H.R., Blanchette P. (2006). Calcium plus vitamin D supplementation and the risk of fractures. N. Engl. J. Med..

[17] Wactawski-Wende J., Kotchen J.M., Anderson G.L., Assaf A.R., Brunner R.L., O’Sullivan M.J., Margolis K.L., Ockene J.K., Phillips L., Pottern L. (2006). Calcium plus vitamin D supplementation and the risk of colorectal cancer. N. Engl. J. Med..

[18] Dean A., Bellgrove M., Hall T., Phan W., Eyles D., Kvaskoff D., McGrath J. (2011). Effects of vitamin D supplementation on cognitive and emotional functioning in young adults—A randomised controlled trial. PLoS One.

[19] Jorde R., Figenschau Y. (2009). Supplementation with cholecalciferol does not improve glycaemic control in diabetic subjects with normal serum 25-hydroxyvitamin D levels. Eur. J. Nutr..

[20] Hendrix I., Anderson P., May B., Morris H. (2004). Regulation of gene expression by the CYP27B1 promoter—Study of a transgenic mouse model. J. Steroid Biochem. Mol. Biol..

[21] Turner A.G., Anderson P.H., Morris H.A. (2012). Vitamin D and bone health. Scand. J. Clin. Lab. Investig..

[22] NHMRC Additional Levels of Evidence and Grades for Recommendations for Developers of Guidelines. https://www.nhmrc.gov.au/_files_nhmrc/file/guidelines/developers/nhmrc_levels_grades_evidence_120423.pdf.

[23] Bjelakovic G., Gluud Lise L., Nikolova D., Whitfield K., Wetterslev J., Simonetti Rosa G., Bjelakovic M., Gluud C. (2011). Vitamin D Supplementation for Prevention of Mortality in Adults. Cochrane Database of Systematic Reviews.

[24] Ford E.S., Zhao G., Tsai J., Li C. (2011). Vitamin D and all-cause mortality among adults in USA: Findings from the national health and nutrition examination survey linked mortality study. Int. J. Epidemiol..

[25] Schöttker B., Haug U., Schomburg L., Köhrle J., Perna L., Müller H., Holleczek B., Brenner H. (2013). Strong associations of 25-hydroxyvitamin D concentrations with all-cause, cardiovascular, cancer, and respiratory disease mortality in a large cohort study. Am. J. Clin. Nutr..

[26] Lappe J.M., Travers-Gustafson D., Davies K.M., Recker R.R., Heaney R.P. (2007). Vitamin D and calcium supplementation reduces cancer risk: Results of a randomized trial. Am. J. Clin. Nutr..

[27] Garland C., Gorham E.D., Mohr S.B., Garland F.C. (2009). Vitamin D for cancer prevention: Global perspective. Ann. Epidemiol..

[28] Grant W.B. (2010). Relation between prediagnostic serum 25-hydroxyvitamin D level and incidence of breast, colorectal, and other cancers. J. Photochem. Photobiol..

[29] Bischoff-Ferrari H.A., Dawson-Hughes B., Staehelin H.B., Orav J.E., Stuck A.E., Theiler R., Wong J.B., Egli A., Kiel D.P., Henschkowski J. (2009). Fall prevention with supplemental and active forms of vitamin D: A meta-analysis of randomised controlled trials. BMJ.

[30] Cranney A., Weiler H.A., O’Donnell S., Puil L. (2008). Summary of evidence-based review on vitamin D efficacy and safety in relation to bone health. Am. J. Clin. Nutr..

[31] Gillespie L., Robertson M., Gillespie W. (2009). Interventions for preventing falls in older people living in the community. Cochrane Database Syst. Rev..

[32] Murad M.H., Elamin K.B., Abu Elnour N.O., Elamin M.B., Alkatib A.A., Fatourechi M.M., Almandoz J.P., Mullan R.J., Lane M.A., Liu H. (2011). The effect of vitamin D on falls: A systematic review and meta-analysis. J. Clin. Endocrinol. Metab..

[33] Scragg R. (2012). Do we need to take calcium with vitamin D supplements to prevent falls, fractures, and death?. Curr. Opin. Clin. Nutr. Metab. Care.

[34] Bischoff-Ferrari H.A., Dietrich T., Orav E.J., Hu F., Zhang Y., Karlson E., Dawson-Hughes B. (2004). Higher 25-hydroxyvitamin D concentrations are associated with better lower-extremity function in both active and inactive persons aged > or =60 y. Am. J. Clin. Nutr..

[35] Stockton K., Mengersen K., Paratz J., Kandiah D., Bennell K. (2011). Effect of vitamin D supplementation on muscle strength: A systematic review and meta-analysis. Osteoporos. Int..

[36] Grandi N.C., Breitling L.P., Brenner H. (2010). Vitamin D and cardiovascular disease: Systematic review and meta-analysis of prospective studies. Prev. Med..

[37] Sokol S.I., Tsang P., Aggarwal V., Melamed M.L., Srinivas V.S. (2011). Vitamin D status and risk of cardiovascular events: Lessons learned via systematic review and meta-analysis. Cardiol. Rev..

[38] Brøndum-Jacobsen P., Benn M., Jensen G.B., Nordestgaard B.G. (2012). 25-Hydroxyvitamin D levels and risk of ischemic heart disease, myocardial infarction, and early death. Arterioscler. Thromb. Vasc. Biol..

[39] Vacek J.L., Vanga S.R., Good M., Lai S.M., Lakkireddy D., Howard P.A. (2012). Vitamin D deficiency and supplementation and relation to cardiovascular health. Am. J. Cardiol..

[40] Parker J., Hashmi O., Dutton D., Mavrodaris A., Stranges S., Kandala N.-B., Clarke A., Franco O.H. (2010). Levels of vitamin D and cardiometabolic disorders: Systematic review and meta-analysis. Maturitas.

[41] Ginde A.A., Scragg R., Schwartz R.S., Camargo C.A. (2009). Prospective study of serum 25-hydroxyvitamin D level, cardiovascular disease mortality, and all-cause mortality in older U.S. adults. J. Am. Geriatr. Soc..

[42] Manaseki-Holland S., Qader G., Isaq Masher M., Bruce J., Zulf Mughal M., Chandramohan D., Walraven G. (2010). Effects of vitamin D supplementation to children diagnosed with pneumonia in Kabul: A randomised controlled trial. Trop. Med. Int. Health.

[43] Urashima M., Segawa T., Okazaki M., Kurihara M., Wada Y., Ida H. (2010). Randomized trial of vitamin D supplementation to prevent seasonal influenza A in schoolchildren. Am. J. Clin. Nutr..

[44] Camargo C.A., Ganmaa D., Frazier A.L., Kirchberg F.F., Stuart J.J., Kleinman K., Sumberzul N., Rich-Edwards J.W. (2012). Randomized trial of vitamin D supplementation and risk of acute respiratory infection in mongolia. Pediatrics.

[45] Martineau A.R., Honecker F.U., Wilkinson R.J., Griffiths C.J. (2007). Vitamin D in the treatment of pulmonary tuberculosis. J. Steroid Biochem. Mol. Biol..

[46] Gibney K.B., MacGregor L., Leder K., Torresi J., Marshall C., Ebeling P.R., Biggs B.-A. (2008). Vitamin D deficiency is associated with tuberculosis and latent tuberculosis infection in immigrants from sub-saharan Africa. Clin. Infect. Dis..

[47] Ginde A.A., Mansbach J.M., Camargo C.A. (2009). Association between serum 25-hydroxyvitamin D level and upper respiratory tract infection in the third national health and nutrition examination survey. Arch. Intern. Med..

[48] Wayse V., Yousafzai A., Mogale K., Filteau S. (2004). Association of subclinical vitamin D deficiency with severe acute lower respiratory infection in Indian children under 5 y. Eur. J. Clin. Nutr..

[49] Aloia J., Li-Ng M. (2007). Re: Epidemic influenza and vitamin D. Epidemiol. Infect..

[50] Sabetta J.R., DePetrillo P., Cipriani R.J., Smardin J., Burns L.A., Landry M.L. (2010). Serum 25-hydroxyvitamin D and the incidence of acute viral respiratory tract infections in healthy adults. PLoS One.

[51] Mitri J., Dawson-Hughes B., Hu F., Pittas A. (2011). Effects of vitamin D and calcium supplementation on pancreatic β cell function, insulin sensitivity, and glycemia in adults at high risk of diabetes: The Calcium and Vitamin D for Diabetes Mellitus (CaDDM) randomized controlled trial. Am. J. Clin. Nutr..

[52] Von Hurst P.R., Stonehouse W., Coad J. (2010). Vitamin D supplementation reduces insulin resistance in South Asian women living in New Zealand who are insulin resistant and vitamin D deficient—A randomised, placebo-controlled trial. Br. J. Nutr..

[53] Mitri J., Muraru M.D., Pittas A.G. (2011). Vitamin D and type 2 diabetes: A systematic review. Eur. J. Clin. Nutr..

[54] Forouhi N.G., Ye Z., Rickard A.P., Khaw K.T., Luben R., Langenberg C., Wareham N.J. (2012). Circulating 25-hydroxyvitamin D concentration and the risk of type 2 diabetes: Results from the European Prospective Investigation into Cancer (EPIC)-Norfolk cohort and updated meta-analysis of prospective studies. Diabetologia.

[55] Afzal S., Bojesen S.E., Nordestgaard B.G. (2013). Low 25-hydroxyvitamin D and risk of type 2 diabetes: A prospective cohort study and metaanalysis. Clin. Chem..

[56] Gloth F., Alam W., Hollis B. (1999). Vitamin D *vs*. broad spectrum phototherapy in the treatment of seasonal affective disorder. J. Nutr. Health Aginig.

[57] Jorde R., Sneve M., Figenschau Y., Svartberg J., Waterloo K. (2008). Effects of vitamin D supplementation on symptoms of depression in overweight and obese subjects: Randomized double blind trial. J. Intern. Med..

[58] Vieth R., Kimball S., Hu A., Walfish P. (2004). Randomized comparison of the effects of the vitamin D3 adequate intake *versus* 100 mcg (4000 IU) per day on biochemical responses and the wellbeing of patients. Nutr. J..

[59] Lansdowne A., Provost S. (1998). Vitamin D3 enhances mood in healthy subjects during winter. Psychopharmacology.

[60] Hujoel P.P. (2013). Vitamin D and dental caries in controlled clinical trials: Systematic review and meta-analysis. Nutr. Rev..

[61] Dietrich T., Joshipura K.J., Dawson-Hughes B., Bischoff-Ferrari H.A. (2004). Association between serum concentrations of 25-hydroxyvitamin D3 and periodontal disease in the US population. Am. J. Clin. Nutr..

[62] Heath K.M., Elovic E.P. (2006). Vitamin D deficiency: Implications in the rehabilitation setting. Am. J. Phys. Med. Rehab..

[63] Schreuder F., Bernsen R.M.D., van der Wouden J.C. (2012). Vitamin D supplementation for nonspecific musculoskeletal pain in non-western immigrants: A randomized controlled trial. Ann. Fam. Med..

[64] Bailey B.A., Manning T., Peiris A.N. (2012). Vitamin D Testing Patterns among Six Veterans Medical Centers in the Southeastern United States: Links with Medical Costs. Military Med..

[65] Peiris A., Bailey B., Manning T. (2008). The Relationship of Vitamin D Deficiency to Health Care Costs in Veterans. Military Med..

[66] Genuis S. (2012). What’s out there making us sick?. J. Environ. Pubic Health.

[67] Church J., Goodall S., Norman R., Haas M. (2011). An economic evaluation of community and residential aged care falls prevention strategies in NSW. NSW Public Health Bull..

[68] Grant W.B., Cross H.S., Garland C.F., Gorham E.D., Moan J., Peterlik M., Porojnicu A.C., Reichrath J., Zittermann A. (2009). Estimated benefit of increased vitamin D status in reducing the economic burden of disease in western Europe. Prog. Biophys. Mol. Biol..

[69] Holick M.F., Binkley N.C., Bischoff-Ferrari H.A., Gordon C.M., Hanley D.A., Heaney R.P., Murad M.H., Weaver C.M. (2011). Evaluation, treatment, and prevention of vitamin D deficiency: An endocrine society clinical practice guideline. J. Clin. Endocrinol. Metab..

[70] Holick M.F., Binkley N.C., Bischoff-Ferrari H.A., Gordon C.M., Hanley D.A., Heaney R.P., Murad M.H., Weaver C.M. (2012). Guidelines for preventing and treating vitamin D deficiency and insufficiency revisited. J. Clin. Endocrinol. Metab..

[71] Pludowski P., Holick M.F., Pilz S., Wagner C.L., Hollis B.W., Grant W.B., Shoenfeld Y., Lerchbaum E., Llewellyn D.J., Kienreich K., Soni M. (2013). Vitamin D effects on musculoskeletal health, immunity, autoimmunity, cardiovascular disease, cancer, fertility, pregnancy, dementia and mortality—A review of recent evidence. Autoimmun. Rev..

[72] Souberbielle J.C., Body J.J., Lappe J.M., Plebani M., Shoenfeld Y., Wang T.J., Bischoff-Ferrari H.A., Cavalier E., Ebeling P.R., Fardellone P. (2010). Vitamin D and musculoskeletal health, cardiovascular disease, autoimmunity and cancer: Recommendations for clinical practice. Autoimmun. Rev..

[73] Grant W.B., Boucher B.J. (2010). Are Hill’s criteria for causality satisfied for vitamin D and periodontal disease. Dermato-Endocrinology.

[74] Hossein-nezhad A., Spira A., Holick M.F. (2013). Influence of vitamin D status and vitamin D3 supplementation on genome wide expression of white blood cells: A randomized double-blind clinical trial. PLoS One.

[75] Durup D., Jørgensen H.L., Christensen J., Schwarz P., Heegaard A.M., Lind B. (2012). A reverse J-shaped association of all-cause mortality with serum 25-hydroxyvitamin D in general practice: The CopD study. J. Clin. Endocrinol. Metab..

[76] Sempos C.T., Durazo-Arvizu R.A., Dawson-Hughes B., Yetley E.A., Looker A.C., Schleicher R.L., Cao G., Burt V., Kramer H., Bailey R.L. (2013). Is there a reverse J-shaped association between 25-hydroxyvitamin D and all-cause mortality?. J. Clin. Endocrinol. Metab..

[77] Rush L., McCartney G., Walsh D., MacKay D. (2013). Vitamin D and subsequent all-age and premature mortality: A systematic review. BMC Public Health.

[78] Tomson J., Emberson J., Hill M., Gordon A., Armitage J., Shipley M., Collins R., Clarke R. (2013). Vitamin D and risk of death from vascular and non-vascular causes in the Whitehall study and meta-analyses of 12,000 deaths. Eur. Heart J..

[79] Zittermann A., Iodice S., Pilz S., Grant W.B., Bagnardi V., Gandini S. (2012). Vitamin D deficiency and mortality risk in the general population: A meta- analysis of prospective cohort studies. Am. J. Clin. Nutr..

